# GEO data mining and TCGA analysis reveal altered branched chain amino acid metabolism in pancreatic cancer patients

**DOI:** 10.18632/aging.202892

**Published:** 2021-04-21

**Authors:** Jun-Yi Li, Fei Sun, Chun-Liang Yang, Hai-Feng Zhou, Min Gao, Qi Zhang, Hui Chen, Peng Zhou, Jun Xiao, Heng Fan

**Affiliations:** 1 Department of Integrated Traditional Chinese and Western Medicine, Union Hospital, Tongji Medical College, Huazhong University of Science and Technology, Wuhan, China; 2 The Center for Biomedical Research, Tongji Hospital, Tongji Medical College, Huazhong University of Science and Technology, Wuhan, China; 3 Department of Endocrinology, Tongji Hospital, Tongji Medical College, Huazhong University of Science and Technology, Wuhan, China; 4 Department of Oncology, Tongji Hospital, Tongji Medical College, Huazhong University of Science and Technology, Wuhan, China; 5 Department of Immunology, School of Medicine, Yangtze University, Jingzhou, China; 6 Department of Urology, Tongji Hospital, Tongji Medical College, Huazhong University of Science and Technology, Wuhan, China

**Keywords:** branched chain amino acids, pancreatic ductal adenocarcinoma, BCAA uptake, data mining

## Abstract

Pancreatic ductal adenocarcinoma (PDAC) is a highly aggressive tumor of the digestive system which has a less than 1% 5-year survival rate. The pathogenesis of PDAC development is incompletely understood. Genetic predisposition, disease history of chronic pancreatitis and diabetes elevate the risk of PDAC while environmental and dietary factors including smoking, alcohol abuse, high fat/protein intake as well as air pollution exacerbate PDAC progression. BCAAs, consisting of leucine, isoleucine and valine are essential amino acids that are obtained from food and play versatile roles in carcinogenesis. Recent studies have demonstrated that BCAA metabolism affects PDAC development but the results are controversial. To explore the possible engagement of BCAA metabolism in PDAC, we took advantage of the GEO and TCGA database and discovered that BCAA uptake is closely related to PDAC development while BCAA catabolism is down-regulated in PDAC tissue. Besides, NOTCH and MYC are differentially involved in BCAA metabolism in tumor and muscle, and enhanced lipid synthesis is independent of BCAA catabolism. Altogether, we highlight BCAA uptake as a promising target for PDAC treatment.

## INTRODUCTION

Recent studies have highlighted the role of metabolic alteration in tumor development [1, 2]. Branched chain amino acids (BCAAs), consisting of leucine, isoleucine and valine, are essential amino acids that can only be acquired from diet rather than de novo synthesized in human body. Following the cellular uptake from circulation, the catabolic pathway is central to BCAA metabolism. Generally speaking, the imported BCAAs are catalyzed into α-keto acids by branched chain amino acid transaminase 1/2 (BCAT 1/2) in a reversible manner and the resulting α-ketoglutarate and glutamate could provide nitrogen for nucleotide biosynthesis and fuel the DNA replication process [3]. Then, the α-keto acids are further decarboxylated and combined with a CoA group under the enzymatic action of branched-chain ketoacid dehydrogenase (BCKDH) complex. Finally, after a series of irreversible enzymatic actions, the succinyl-CoA and acetyl-CoA are generated to take part in the tricarboxylic acid (TCA) cycle and serve as a carbon source [3, 4]. Free, untransformed intracellular BCAAs are potent stimulators of mTORC1 signaling pathway activation, which leads to enhanced protein synthesis [5, 6]. All these characteristics of BCAA metabolism make it a potential candidate for tumor intervention.

Indeed, BCAA metabolism was found to be critically involved in the progression of multiple cancer types [7–9], especially for the pancreatic ductal adenocarcinoma (PDAC) with controversial results being reported [10, 11]. Some research has shown that BCAA uptake is down-regulated in PDAC tissue [10], while other research has demonstrated an elevated BCAA uptake along with enhanced expression of corresponding solute carrier (SLC) transporters (SLC7A5, SLC1A5, SLC3A2, SLC43A1, SLC43A2) [12]. In Lee’s study, BCAT2 was found elevated in mouse PDAC model and human patients. Pancreatic tissue specific knockout of BCAT2 impeded progression of pancreatic intraepithelial neoplasia (PanIN) in LSL-KrasG12D/+ mice [13]. However, Mayers and colleagues found decreased SLC7A5, BCAT1, BCAT2 and BCKDH expression in PDAC tissue, and loss of BCAT2 does not affect PDAC tumor formation [14]. Similarly, PDAC and non–small cell lung carcinoma (NSCLC) obtain BCAAs as a nitrogen source, whereas deletion of BCAT1 and BCAT2 could prevent NSCLC tumor formation *in vivo* but has limited effect on PDAC tumor growth [10, 14]. Nonetheless, the increased plasma BCAA level associates with worse prognosis of pancreatic cancer, and BCAT2 expression in PDAC tissue was independently correlated to high mortality rate [11]. Lines of evidence consistently indicated an elevated level of BCAA in the plasma of PDAC patients, which may result from accelerated muscle wasting in individuals suffering from cachexia [10]. Despite the fact that the mechanisms underlying how PDAC cells utilize the BCAAs remain enigmatic, lipid biosynthesis that is pivotal for unlimited proliferation and invasive metastasis of cancer cells is gaining much attention [12, 15]. Adipocyte and lipid metabolism are also responsible for the drug resistance in cancer [16]. BCAA degrading enzymes promote lipogenesis pathway but do not affect glucose metabolism and mitochondrial respiration [12]. In addition, BCKDHA knockdown significantly inhibited fatty acid synthesis while having no effect on levels of tricarboxylic acid cycle (TCA) intermediates or the oxygen consumption rate (OCR) [12].

Taken together, the BCAA uptake, expression of BCAA metabolic enzymes and their functional effect in PDAC tumor cell progression are still under debate. To unveil the role of BCAA metabolism in human PDAC and its relationship with lipid metabolism, we took advantage of three human PDAC datasets acquired from public GEO database to explore the alteration of BCAA metabolism related genes, and the TCGA database to carry out survival analysis. These results comprehensively provide clinicians and researchers with a grand landscape of BCAA metabolism in PDAC development.

## RESULTS

### Identification of differentially expressed genes (DEGs) between PDAC and normal pancreatic tissue

**Figure 1:**
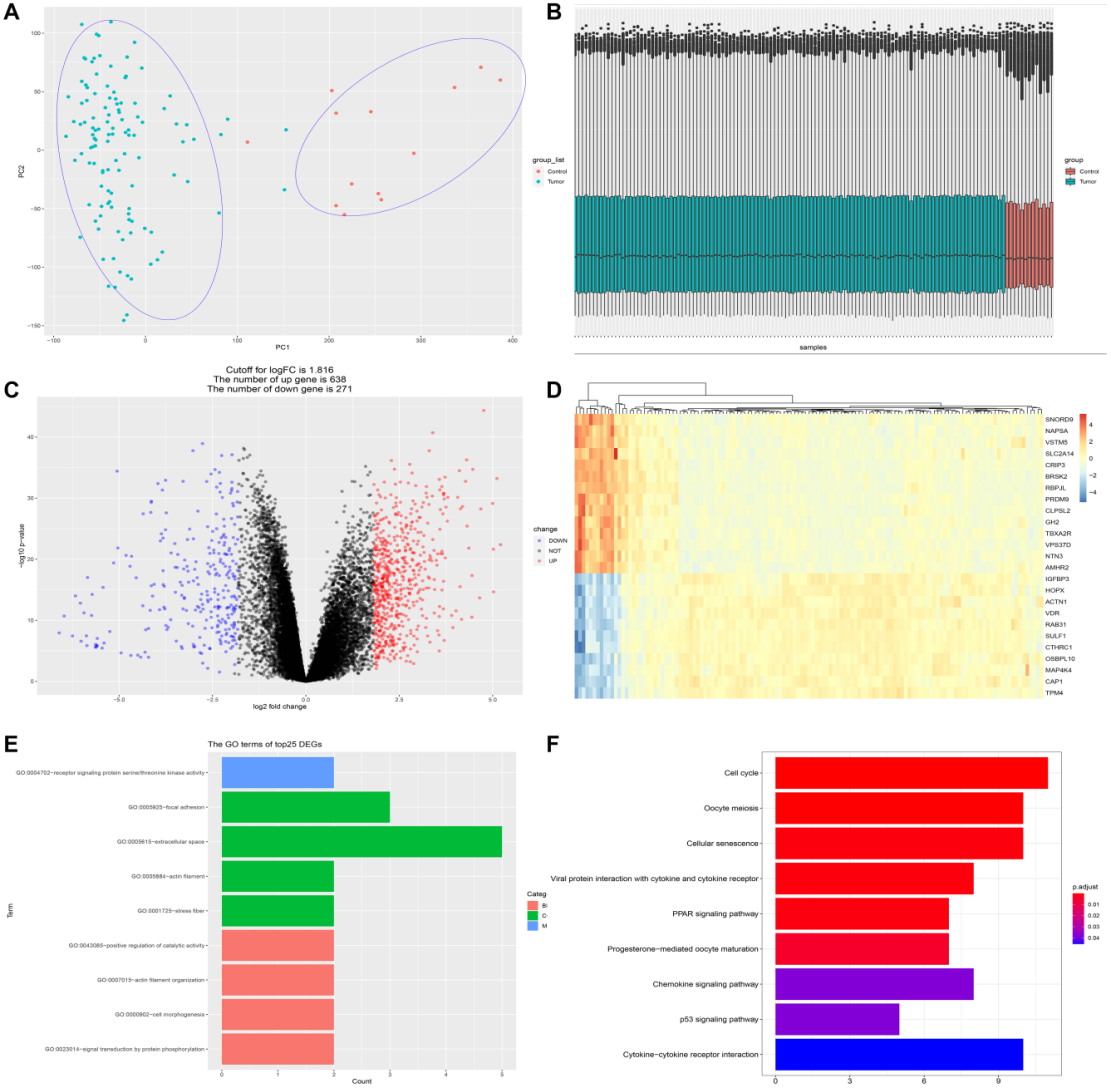
**Identification of differentially expressed genes (DEGs) between PDAC and normal pancreatic tissue.** (**A**) PCA analysis discriminates PDAC group (*n* = 118) from control group (*n* = 13). (**B**) The dataset (GSE62165) conformed to sample homogeneity. (**C**) A total of 909 DEGs were obtained in GSE62165, among which 271 genes were down-regulated and 638 genes up-regulated. (**D**) The top 25 DEGs exhibited by heatmap. (**E**) The GO terms of the top 25 DEGs. GO analysis of top 25 DEGs is related to cell skeleton components (extracellular space, actin filament, stress fiber, etc.) and protein phosphorylation events. (**F**) The KEGG analysis of the top 25 DEGs. The most significant pathways are cell cycle regulation, cellular senescence, PPAR signaling, P53 signaling, chemokine signaling and cytokine-cytokine receptor interaction.

GSE62165, comprised of 118 PDAC and 13 normal control pancreatic samples, was used to identify the DEGs between PDAC and normal tissue. The dataset conformed to sample homogeneity and PCA analysis could clearly discriminate PDAC group from control group (Figure 1A–1B). A total of 909 DEGs were obtained in GSE62165 (the cut-off value of logFC is 1.816), among which 271 genes were down-regulated and 638 genes up-regulated (Figure 1C). The top 25 DEGs exhibited by heatmap were displayed in Figure 1D. We further analyzed the functional enrichment of the top 25 DEGs (Figure 1E–1F). GO analysis showed that cell skeleton components and protein phosphorylation events are closely related to their effect, and KEGG analysis indicates the involvement of pathways like cell cycle regulation, cellular senescence, PPAR signaling, P53 signaling, chemokine signaling and cytokine-cytokine receptor signaling pathway, which are all well-appreciated in tumor development. Therefore, this set of data is eligible for further investigation.

### Altered BCAA uptake in PDAC tissue

**Figure 2:**
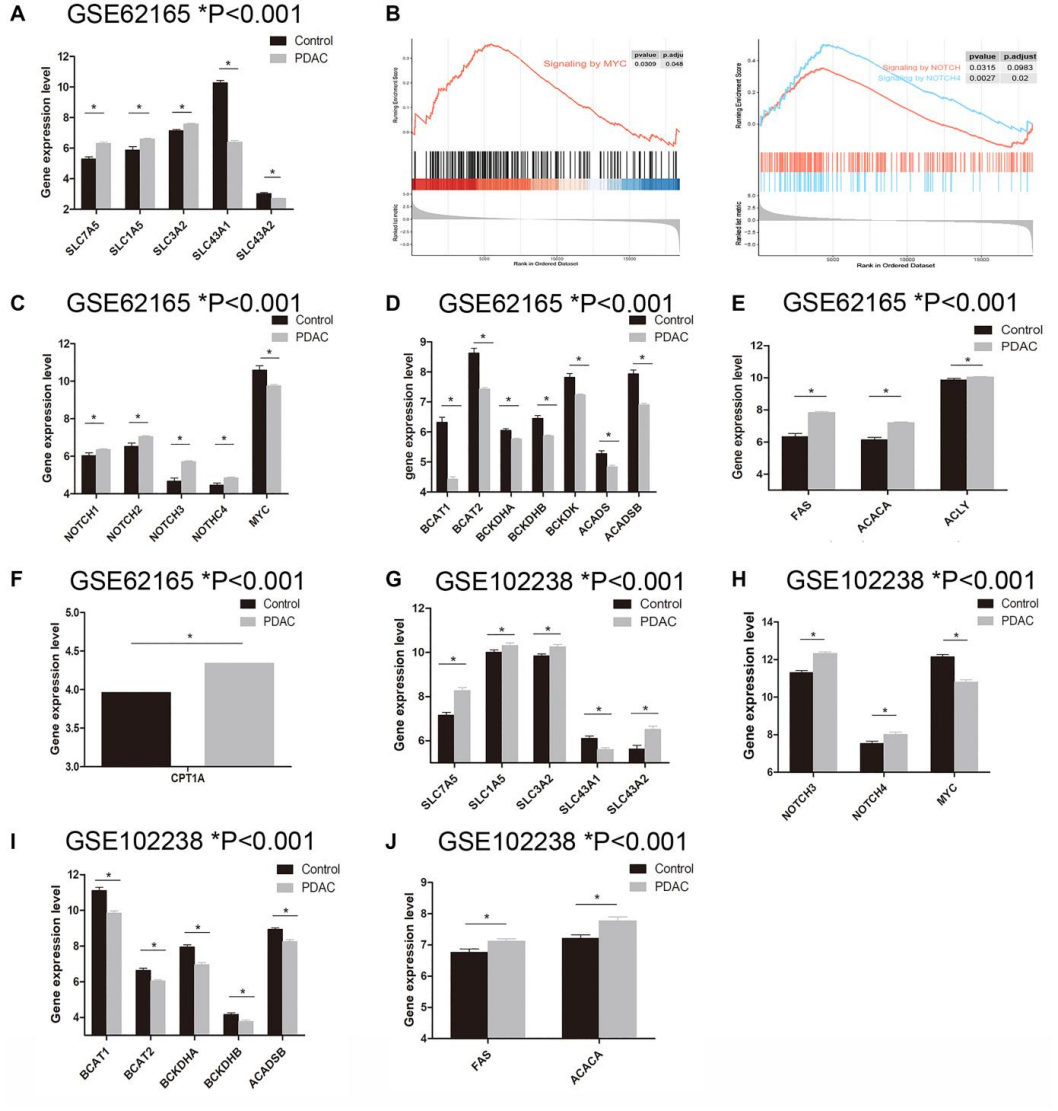
**Examination of genes related to BCAA and fatty acid metabolism in PDAC tissue. A**–**F**: Data mining in GSE62165 dataset. (**A**) The expression level of SLC carriers (SLC7A5, SLC1A5, SLC3A2, SLC43A1 and SLC43A2) between PDAC and normal tissue. (**B**) GSEA analysis revealed enriched MYC and NOTCH signaling pathway in PDAC tissue. (**C**) The expression level of MYC and NOTCH (NOTCH1-4) between PDAC and normal tissue. (**D**) BCAA catabolism related genes BCAT1, BCAT2, BCKDHA, BCKDHB, BCKDK, ACADS and ACADSB were found significantly down-regulated in PDAC group. (**E**) The expression of lipogenesis related enzymes including FAS, ACACA and ACLY were significantly up-regulated in PDAC tissue. (**F**) CPT1A, the rate-limiting enzyme for fatty acid oxidation was notably upregulated in PDAC tissue. **G**–**J**: Data mining in GSE102238 dataset. (**G**) The expression level of SLC carriers (SLC7A5, SLC1A5, SLC3A2, SLC43A1 and SLC43A2) between PDAC and adjacent normal tissue. (**H**) NOTCH3 and NOTCH4 were upregulated in PDAC tissue while MYC was down-regulated. (**I**) Gene expression of BCAT1, BCAT2, BCKDHA, BCKDHB and ACADSB were decreased in PDAC tissue. (**J**) FAS and ACACA were increased in PDAC tissue.

**Figure 3:**
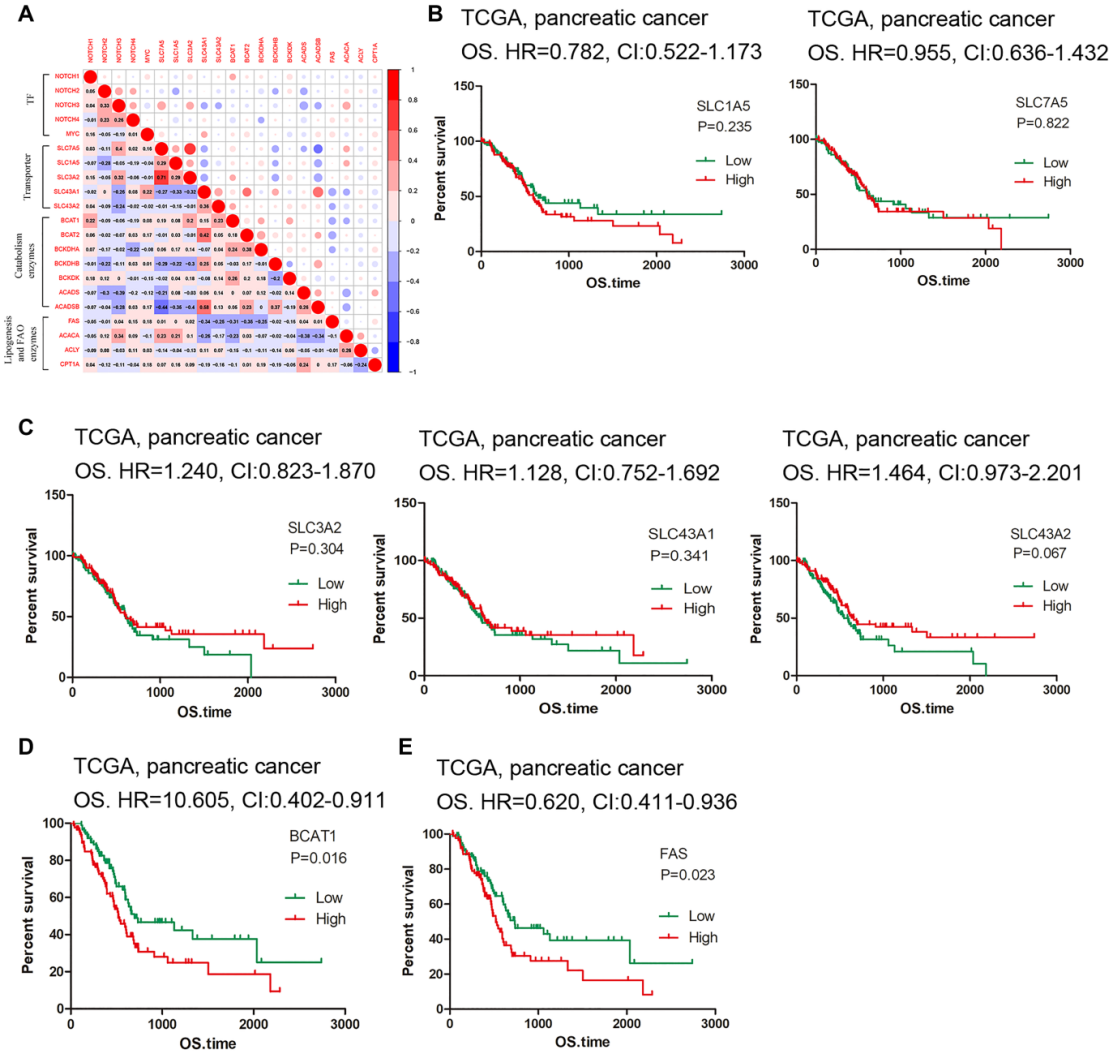
**Correlation and survival analysis of the hub genes.** (**A**) Matrix showing degree of correlation among the selected genes. (**B**–**C**) Survival curves of SLC carriers. (**D**–**E**) High expression of BCAT1 and FAS indicated low survival rate.

Using GSE62165 as the discovery set, we evaluated the BCAA metabolic alteration in PDAC patients. Studies have reported that SLC7A5, SLC1A5, SLC3A2, SLC43A1 and SLC43A2 are the major BCAA transporters in tumor cells [12, 17]. To dissect which one plays a central role in BCAA transportation in pancreatic cancer, we assessed the expression level of these SLC carriers between PDAC and normal tissue. The gene expression level of SLC7A5 (6.316 ± 0.088 vs 5.299 ± 0.129, *P* = 0.0002), SLC1A5 (6.593 ± 0.051 vs 5.884 ± 0.217, *P* < 0.0001) and SLC3A2 (7.588 ± 0.042 vs 7.143 ± 0.076, *P* = 0.0008) were found notably up-regulated while SLC43A1 (6.395 ± 0.104 vs 10.290 ± 0.140, *P* < 0.0001) and SLC43A2 (2.723 ± 0.013 vs 3.036 ± 0.068, *P* < 0.0001) are considerably down-regulated in PDAC group in comparison with control group (Figure 2A). MYC and NOTCH, suggested to directly regulate the expression of SLC transporters, are two key transcriptional regulators that promote the uptake of essential amino acids [18–20]. Gene set enrichment analysis (GSEA) revealed enhanced activation of MYC and NOTCH signaling pathway in PDAC (Figure 2B). Interestingly, gene expression level of MYC in PDAC group is lower than that of control group (9.758 ± 0.062 vs 10.600 ± 0.226, *P* < 0.0001) (Figure 2C), while NOTCH1 (6.354 ± 0.042 vs 6.046 ± 0.141, *P* = 0.023), NOTCH2 (7.051 ± 0.047 vs 6.536 ± 0.165, *P* = 0.0008), NOTCH3 (5.721 ± 0.044 vs 4.676 ± 0.164, *P* < 0.0001), NOTCH4 (4.858 ± 0.038 vs 4.473 ± 0.105, *P* = 0.0015) are higher in PDAC group (Figure 2C). Although NOTCH1-4 are all up-regulated in PDAC cells, correlation analysis demonstrated that only NOTCH3 expression level is in moderate positive correlation with SLC7A5 and SLC3A2, r = 0.399 and r = 0.319, respectively (Figure 3A). These data hint that NOTCH and MYC are mainly responsible for BCAA uptake in PDAC tumor cells.

### Down-regulated BCAA catabolism and enhanced fatty acid metabolism in PDAC tissue

BCAA catabolism related genes BCAT1, BCAT2, BCKDHA, BCKDHB, BCKDK, ACADS and ACADSB were found significantly down-regulated in PDAC group (Figure 2D). As known, the fatty acid biosynthesis enzymes such as acetyl-CoA carboxylase (ACC), fatty-acid synthase (FASN) and ATP-citrate lyase (ACLY) were reported to be greatly increased in various tumors [12, 21, 22]. Meanwhile, BCAA catabolism was shown to inhibit fatty acid oxidation (FAO) while promoting lipid accumulation [23, 24]. Compared to normal tissue, the expression of lipogenesis related enzymes including FAS (7.838 ± 0.055 vs 6.345 ± 0.193, *P* < 0.0001), ACACA (7.213 ± 0.041 vs 6.142 ± 0.145, *P* < 0.0001) and ACLY (10.050 ± 0.024 vs 9.873 ± 0.083, *P* = 0.020) are significantly up-regulated in PDAC tissue (Figure 2E). Of note, the FAO also seems enhanced in pancreatic cancer. Expression level of CPT1A, the rate-limiting enzyme of FAO, is notably upregulated in PDAC tissue comparison with that of normal tissue (4.342 ± 0.044 vs 3.965 ± 0.087, *P* = 0.006) (Figure 2F). Overall, the BCAA catabolism genes are down-regulated while the lipid metabolism related genes are up-regulated in the PDAC tissue.

### Validation set

In order to validate our findings, we used GSE102238 dataset to confirm above results. GSE102238 contains 100 cases of paired PDAC tissue and the adjacent normal tissue. In PDAC tissue, gene expression level of SLC7A5 (8.269 ± 0.148 vs 7.154 ± 0.233, *P* < 0.001), SLC1A5 (10.320 ± 0.109 vs 10.010 ± 0.101, *P* = 0.040), SLC3A2 (10.260 ± 0.106 vs 9.846 ± 0.087, *P* = 0.003) and SLC43A2 (6.516 ± 0.144 vs 5.625 ± 0.167, *P* = 0.0001) are similarly up-regulated and SLC43A1 (5.607 ± 0.082 vs 6.105 ± 0.104, *P* < 0.001) is down-regulated (Figure 2G). Although gene expression data of NOTCH1 and NOTCH2 are absent from this dataset, we found that NOTCH3 (12.330 ± 0.081 vs 11.300 ± 0.110, *P* < 0.001) and NOTCH4 (8.018 ± 0.120 vs 7.539 ± 0.127, *P* = 0.007) expression level in PDAC tissue are upregulated while MYC (10.800 ± 0.114 vs 12.150 ± 0.126, *P* < 0.001) is down-regulated (Figure 2H). Gene expression level of BCAT1 (9.841 ± 0.122 vs 11.11 ± 0.170, *P* < 0.001), BCAT2 (6.051 ± 0.065 vs 6.643 ± 0.107, *P* < 0.001), BCKDHA (6.955 ± 0.119 vs 7.944 ± 0.131, *P* < 0.001), BCKDHB (3.777 ± 0.074 vs 4.166 ± 0.096, *P* < 0.001) and ACADSB (8.257 ± 0.095 vs 8.933 ± 0.086, *P* < 0.001) are decreased in PDAC tissue (Figure 2I). And for lipogenesis related genes, FAS (7.118 ± 0.081 vs 6.763 ± 0.105, *P* = 0.009) and ACACA (7.779 ± 0.120 vs 7.214 ± 0.109, *P* < 0.001) are increased in PDAC tissue (Figure 2J). Unfortunately, the expression data of CPT1A is absent from this dataset. Nevertheless, the comparative results between PDAC tissue and adjacent tissue corroborate the findings obtained from PDAC and normal tissue samples.

### Correlation and survival analysis

To further understand the relationship between BCAA catabolism and fatty acid biosynthesis/oxidation, correlation analysis was conducted. ACACA gene expression is in moderate negative correlation with ACADS and ACADSB expression (r = -0.383, r = -0.344) and FAS was in moderate negative correlation with BCAT1 and BCAT2 (r = -0.305, r = -0.348) (Figure 3A). The rest of genes displays no obvious correlation; thus, contrary to what has been anticipated, there does not exist a positive link between BCAA catabolism and lipid biosynthesis. Finally, we used the pancreatic cancer dataset in TCGA for survival analysis. We first check the BCAA carriers. Although none of them reached statistic difference, the tendency showed that high expression of SLC1A5 and SLC7A5 is linked to low survival rate while high expression of SLC3A2, SLC43A1 and SLC43A2 is related to high survival rate (Figure 3B–3C). Additionally, we found that high expression of BCAT1 (HR = 0.605, CI: 0.402-0.911, *P* = 0.016) and FAS (HR = 0.620, CI: 0.411-0.936, *P* = 0.023) indicates low survival rate (Figure 3D–3E), while among the rest of associated genes examined, none of them displayed statistical significance (data not shown). These results reflected the flexibility of the BCAA transporter usage, and their role in predicting PDAC prognosis deserves large sample investigations.

### Experimental verification

**Figure 4:**
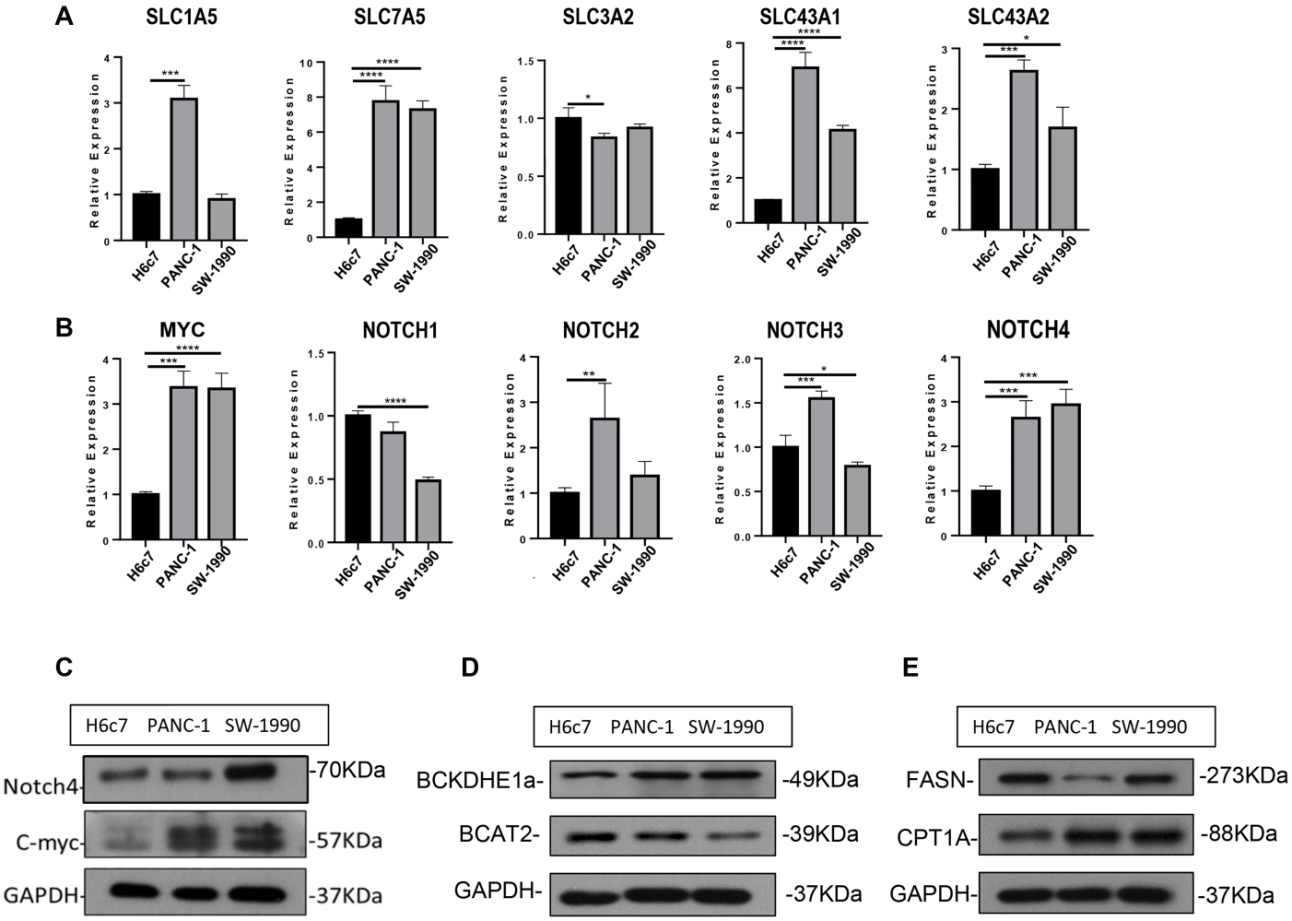
**Experimental verification of the bioinformatic results.** (**A**) mRNA expression of SLC carriers in H6c7, PANC-1 and SW-1990 cell line. (**B**) mRNA expression of MYC and NOTCH in H6c7, PANC-1 and SW-1990 cell line. (**C**–**E**) Protein expression of MYC, NOTCH4, BCAT2, CPT1A and FASN in H6c7, PANC-1 and SW-1990 cell line.

To further verify the aforementioned results, we took advantage of three distinct human pancreatic duct epithelial cell lines: the HPDE6-E6E76c7 (H6c7) cell line was immortalized from normal human pancreatic duct epithelial (HPDE) cells, PANC-1 derived from a human carcinoma of exocrine pancreas, and SW-1990 was established in 1978 from a spleen metastasis of a grade II pancreatic adenocarcinoma. SW-1990 possesses the highest degree of malignancy followed by PANC-1, while H6c7 could serve as a normal control. Regarding the BCAA transporters, SLC7A5 exhibited considerably increased expression level in both PANC-1 and SW-1990 cells. SLC1A5, SLC3A2 and SLC43A2 were mostly enriched in PANC-1 cell line, whereas SLC43A1 was slightly decreased in both PANC-1 and SW-1990 cells (Figure 4A). These results corroborated the flexibility of BCAA transporter usage, which brought challenge for therapies aimed at limiting BCAA uptake in PDAC. The similar flexibility was also observed in the upstream regulator NOTCH family proteins: as NOTCH4 was highly expressed in both PANC-1 and SW-1990 cells, NOTCH2 and NOTCH3 were highly expressed only in PANC-1 while NOTCH1 was enriched in H6c7 cell line. Contrary to the bioinformatic analysis, MYC displayed an increased expression level in both PANC-1 and SW-1990 cell lines (Figure 4B), and the enhanced expression of MYC and NOTCH4 ICD (intracellular domain) was confirmed at the protein level (Figure 4C). Consistently, the decreased BCAT2 expression indicated a diminished BCAA catabolism while the elevated CPT1A implied strengthened fatty acid oxidation; however, we were unable to detect an increase of FASN expression in PANC-1 and SW-1990 cell lines (Figure 4D–4E).

### MYC mainly regulates BCAA uptake in myocyte of PC patients

**Figure 5:**
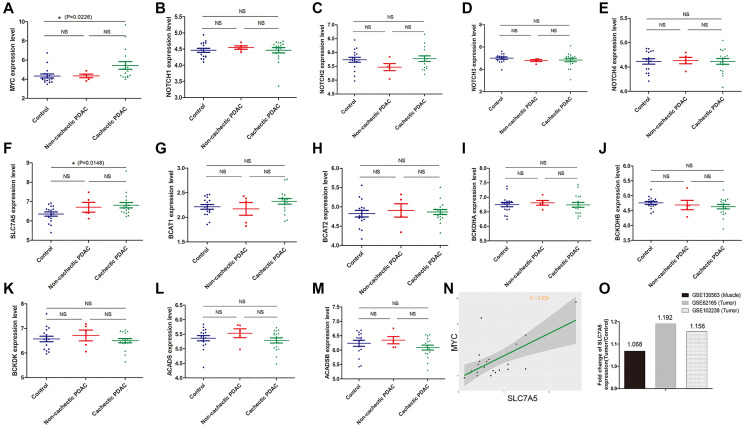
**MYC but not NOTCH mainly regulates BCAA uptake in myocyte of PDAC patients.** (**A**–**O**): Data mining in GSE130563 dataset. (**A**–**E**) Gene expression of NOTCHs did not vary between groups while MYC was significantly up-regulated in muscles of cachectic PDAC patients. (**F**) Most of the SLC transporters exhibited no change except for SLC7A5 whose expression was slightly up-regulated in muscles of cachectic PDAC patients. (**G**–**M**) Gene expression of BCAA catabolism related genes in muscle. (**N**) In GSE130563 dataset, correlation analysis showed that SLC7A5 expression was in moderate correlation with MYC expression in muscle of PDAC patients (r = 0.639). (**O**) The relative fold-change of SLC7A5 expression in GSE62165 (tumor) and GSE102238 (tumor) were higher than that in GSE130563 (muscle).

Muscle is another important place where BCAAs are produced or deposited [25, 26]. To unveil the BCAA metabolism in muscle of pancreatic cancer patients, GSE130563 dataset was additionally applied to do further investigation. We discovered that gene expression of NOTCHs does not vary between groups while MYC (5.428 ± 0.394 vs 4.333 ± 0.214, *P* = 0.023) expression in muscles of cachectic PDAC patients was significantly up-regulated (Figure 5A–5E). Most of the SLC transporters exhibit no change between groups, except for SLC7A5 (6.813 ± 0.638 vs 6.355 ± 0.109, *P* = 0.015) whose expression in muscles of cachectic PDAC patients is slightly up-regulated compared to that of normal control (Figure 5F). At the same time, BCAA catabolism related genes exhibited no significant difference (Figure 5G–5M). Correlation analysis showed that SLC7A5 expression was in moderate correlation with MYC expression in muscle of PDAC patients (r = 0.639) (Figure 5N). Since SLC7A5 is up-regulated both in PDAC tissue and muscle of PDAC patients, fold-change (relative to normal counterparts) of SLC7A5 expression in muscle and tumor was calculated to determine which one potentially acquires more BCAAs from circulation. These results indicate that the relative fold-change of SLC7A5 expression in GSE62165 (tumor) and GSE102238 (tumor) are higher than that in GSE130563 (muscle) (Figure 5O), which means PDAC tumor cells might be more capable than myocytes regarding to BCAA uptake. Thus, distinct from tumor cells, MYC instead of NOTCH is a possible upstream transcriptional regulator of SLC transporters in muscle. During PDAC cancer development, cachectic condition in particular, pancreatic tissue robustly enhance the BCAA absorptive activity with only minor change in skeletal muscles.

## Discussion and Conclusion

Metabolic pathways are increasingly being appreciated in tumor intervention [27, 28]. With the aim to clarify the relationship between BCAA metabolism and PDAC development, we utilize the open accessed databases (GEO and TCGA) to address some of the most concerned issues. Intriguingly, we found BCAA uptake elevated while BCAA catabolism decreased in PDAC tissue, implying that the tumor-favoring effect is a major consequence of free BCAAs which act as potent stimulators of mTOR signaling pathway. Lipid metabolism including fatty acid biosynthesis and beta oxidation is enhanced in PDAC, but no positive associative link between lipid metabolism and BCAA catabolism is observed. Most likely, BCAAs does not serve as a supplying source for lipid metabolism in pancreatic cancer, which is consistent with a down-regulated BCAA catabolic pathway.

Therefore, the key to BCAA targeted therapy for PDAC falls into blockade of BCAA uptake. NOTCH and MYC are critical for the transcription of SLC carriers that are responsible for BCAA transportation [18–20]. Notably, NOTCH appears to be important in PDAC tissue given its higher expression and the positive correlation with SLC7A5 and SLC3A2. On the other hand, in skeletal muscle, MYC plays a pivotal role but the only identified differentially expressed BCAA transporter, SLC7A5, shows merely subtle change. BCAA is substantial to the maintenance of muscle mass [26, 29]. During cancer-induced cachexia, muscle proteins would degrade and release BCAAs into peripheral blood [29]. However, it seems that circulating BCAAs prefer to flux into PDAC tissue rather than recycling back to the muscle, which possibly explain the unrestrained tumor progression. This discovery is in consistent with another study suggesting that loss of skeletal muscle, but not adipose tissue, is associated with shorter survival time of patients with advanced pancreatic cancer [30]. Overall, targeting on specific SLC carriers or NOTCH signaling pathway represents a promising strategy in PDAC treatment, and may also help to ameliorate the cachectic syndrome.

The shortcomings of this study stem from the relatively small sample size and lack of enough experimental data. For instance, the possible role of BCAA metabolic enzymes serving as PDAC prognostic biomarkers and the correlation with survival rate are inadequately revealed. Nonetheless, our work provides a meaningful view on the relationship between BCAA metabolism and PDAC development, which sheds novel light on future clinical translational studies.

## MATERIALS AND METHODS

### Gene expression data downloaded from GEO

Using the keywords “pancreatic ductal adenocarcinoma GEO accession” to search on the GEO database, the gene expression profiles of GSE62165 and GSE102238 (Cheng et al, unpublished data, 2015) were downloaded. The GSE62165 profile consisted of 118 PDAC samples and 13 corresponding normal tissues. GSE102238 contained 100 cases of paired PDAC tissue and the adjacent normal tissue. GSE130563 dataset included muscle samples of pancreatic cancer patients. The R software package was applied to process the downloaded files and to reject the unqualified data. The data were calibrated, standardized, and log2 transformed.

### Principal component analysis (PCA) and correlation analysis

Principal component analysis (PCA) was performed with R package to the degree of aggregation of pancreatic samples. Correlation values were calculated via Speaman's correlation to evaluate the relationship between the selected genes.

### Identification of differentially expressed genes (DEGs

The downloaded platform and series of matrix file(s) were converted using the R software. The ID corresponding to the probe name was converted into an international standard name for genes (gene symbol) and saved in a txt file. Genes with the parameter of false discovery rate (FDR) below 0.05 (a corrected *P*-value < 0.05) and log fold-change (log2FC) values >2 were considered as differentially expressed genes (DEGs).

### Gene ontology (GO) and KEGG enrichment analysis

All DEGs were mapped to GO terms in the Gene Ontology database. Gene numbers were calculated for every term and GO terms with an FDR < 0.05 and *P*-value < 0.05 were defined as significantly enriched terms. KOBAS online analysis database was employed for KEGG pathway analysis of DEGs. Significantly enriched metabolic pathways or signal transduction pathways in DEGs were identified by comparing them with the whole genome background. Pathways meeting the condition of an FDR < 0.05 (*P*-value < 0.05) were considered statistically significant.

### Gene set enrichment analysis

GSEA software (Broad Institute) was used to determine the statistically significant gene sets in the comparison between different subgroups. Then, annotated gene sets from the HALMARK collection v7.1 were used as enrichment input. Significantly enriched gene sets were defined as gene sets with a normalized enrichment score NES) > 1 and FDR< 0.05.

### Cell culture

Human pancreatic duct epithelial cell lines H6c7, pancreatic carcinoma cell lines PANC-1 and SW-1990 (ATCC) were cultured in RPMI-1640 medium (Gibco) containing 10 mM D-glucose supplemented with 10% FBS (Gibco) and 1% penicillin/streptomycin in a 5% CO_2_ incubator at 37°C. All cell lines were routinely tested for negative mycoplasma contamination using Mycoplasma Detection Kit (Lonza).

### Quantitative real-time PCR (qRT-PCR)

Total RNA was extracted with the Trizol reagent (Takara, Japan). An aliquot containing 1 μg of total RNA was reverse transcribed into cDNA using a PrimeScript™ RT Master Mix (Takara, Japan), and the cDNA was further amplified by the SYBR Green Real-time PCR Master Mix (TaKara, Japan) in the ABI Prism 7500 Sequence Detection System (Applied Biosystems, South San Francisco, USA). The following primers were used: MYC forward 5′-CCTGG TGCTC CATGA GGAGA C-3′, and reverse 5′-CAGAC TCTGA CCTTT TGCCA GG-3′; NOTCH1 forward 5′-GGTGA ACTGC TCTGA GGAGA TC-3′, and reverse 5′-GGATT GCAGT CGTCC ACGTT GA-3′; NOTCH2 forward 5′-GTGCC TATGT CCATC TGGAT GG-3′, and reverse 5′-AGACA CCTGA GTGCT GGCAC AA-3′; NOTCH3 forward 5′-TACTG GTAGC CACTG TGAGC AG-3′, and reverse 5′-CAGTT ATCAC CATTG TAGCC AGG-3′; NOTCH4 forward 5′-TTCCA CTGTC CTCCT GCCAG AA-3′, and reverse 5′-TTCCA CTGTC CTCCT GCCAG AA-3′; solute carrier family 1 member 5 (SLC1A5) forward 5′-TCCTC TTCAC CCGCA AAAAC CC-3′, and reverse 5′-CCACG CCATT ATTCT CCTCC AC-3′; solute carrier family 3 member 2 (SLC3A2) forward 5′-CCAGA AGGAT GATGT CGCTC AG-3′, and reverse 5′-GAGTA AGGTC CAGAA TGACA CGG-3′; solute carrier family 7 member 5 (SLC7A5) forward 5′-GCCAC AGAAA GCCTG AGCTT GA-3′, and reverse 5′-ATGGT GAAGC CGATG CCACA CT-3′; solute carrier family 43 member 1 (SLC43A1) forward 5′-GATGC TGGAG TACCT TGTGA CTG-3′, and reverse 5′-CAGGT GAGAA GGCAC AACAG CT-3′; solute carrier family 43 member 2 (SLC43A2) forward 5′-TCGGC AGTCA CCTTT CCAGG AA-3′, and reverse 5′-GAAGC AGTTG AGGAA AACCA GCC-3′; β-actin forward 5′-CATTG CTGAC AGGAT GCAGA AGG-3′, and reverse 5′-TGCTG GAAGG TGGAC AGTGA GG-3′. The relative expression level of each gene was calculated with the 2˗ΔΔCt method as previously reported and normalized to the β-actin expression level.

### Western blot analysis

Cell lysates were prepared using the radio-immunoprecipitation assay (RIPA) buffer (Servicebio, Wuhan, China) containing a protease inhibitor cocktail (Roche, IN, USA). Western blot analysis of target proteins was conducted as described using the indicated primary antibodies, followed by probing to the corresponding HRP-conjugated secondary antibody. The reactive bands were visualized using ECL plus reagents (Servicebio, Wuhan, China). GAPDH was used as an internal control to analyze the results. The detailed information about the antibodies was as following:

Anti-BCKDE1a (#90198), anti-BCAT2 (#79764), anti-NOTCH4 (#2423), anti-c-myc (#14819) and anti-fatty acid synthase (FAS, #3180) antibodies were obtained from the Cell Signaling Technology (Danvers, MA, USA). Anti-CPT1a antibody (#15184-1) was purchased from Proteintech Group (Wuhan, China). Antibody against GAPDH was purchased from Santa Cruz Biotechnology (Santa Cruz, CA, USA).

### Survival analysis of the hub genes

Kaplan-Meier (KM) Plotter, one of the publicly available datasets for cancer microarray with clinical annotations, was employed to evaluate the prognostic value of the indicated genes in PDCA. PDCA patients were divided into high and low expression groups using the median level. The selected genes were inputted as a query and the data were collected for overall survival (OS) analysis. The log rank p values and the hazard ratio (HR) with 95% confidence intervals (95% CI) were calculated and displayed.

### Statistics

The statistical significance between the tumor and normal groups was calculated by Student's *t* test. The statistical analysis and illustration were performed by GraphPad Prism 6 (San Diego, CA, USA) and R software (Version 3.5.1). Adjusted *p* value < 0.05 and log2FC > 2 were set up as the significant cut-off for DEGs only.
